# A 3D-Printed Scaffold for Repairing Bone Defects

**DOI:** 10.3390/polym16050706

**Published:** 2024-03-05

**Authors:** Jianghui Dong, Hangxing Ding, Qin Wang, Liping Wang

**Affiliations:** Guangxi Engineering Research Center of Digital Medicine and Clinical Translation, School of Intelligent Medicine and Biotechnology, Guilin Medical University, Guilin 541199, China; djh1028@163.com (J.D.); hangxing-ding@163.com (H.D.); qinwang_2021@163.com (Q.W.)

**Keywords:** 3D-printed scaffold, ceramic material, polycaprolactone composite scaffolds, gelatin composite scaffolds

## Abstract

The treatment of bone defects has always posed challenges in the field of orthopedics. Scaffolds, as a vital component of bone tissue engineering, offer significant advantages in the research and treatment of clinical bone defects. This study aims to provide an overview of how 3D printing technology is applied in the production of bone repair scaffolds. Depending on the materials used, the 3D-printed scaffolds can be classified into two types: single-component scaffolds and composite scaffolds. We have conducted a comprehensive analysis of material composition, the characteristics of 3D printing, performance, advantages, disadvantages, and applications for each scaffold type. Furthermore, based on the current research status and progress, we offer suggestions for future research in this area. In conclusion, this review acts as a valuable reference for advancing the research in the field of bone repair scaffolds.

## 1. Introduction

Tumor removal, deformities, sports injuries, and infections can lead to bone defects [1]. There are more than 6.5 million cases of bone defects in the United States each year [1,2]. Bone grafting is the most common method to treat bone defects [3]. Globally, there are two million bone grafts performed annually [4]. The “gold standard” for treating bone defects is autologous bone transplantation in the clinic [5]. However, the source of bone grafts is limited, and the second operation may cause additional pain for patients. Moreover, allogeneic bone transplantation can produce an immune response [6,7,8]. Allogeneic bone graft substitutes possess different osteoinductive and osteoconductive properties and a lower osteogenic potential than do autologous bone grafts [9]. Repairing large, severe bone defects is challenging due to the high risk of delayed healing or even non-healing [10].

The development of bone tissue engineering has opened up new avenues for treating bone defects [11,12]. Bone tissue engineering scaffolds can fill the defect, restore the anatomical structure, promote the formation of new blood vessels and bone tissue, and ultimately achieve the regenerative repair of diseased bone tissue [13,14,15]. The three elements of bone tissue engineering are seed cells, growth factors, and scaffold materials [16], among which scaffold materials play a role in mimicking the extracellular matrix and providing a suitable microenvironment for cell growth and differentiation. A good bone tissue engineering scaffold exhibits biocompatibility, osteoconductivity, osteoinductivity, degradability, and mechanical properties similar to those of the defective bone [17]. In repairing critical-size bones, bone scaffolds can support the formation of new tissue cells [18]. Bone scaffolds with appropriate degradability can effectively promote the healing of critical-size bones.

The traditional methods to fabricate tissue culture scaffolds include solvent casting, particulate leaching [19], CO_2_ gas-foaming [20], phase separation [21], freeze-drying methods, etc. [22]. However, using these methods, it is not easy to adjust the porosity, pore size, pore distribution, and interconnectivity. In addition, bone scaffolds should possess good osteoconductivity, osteoinductivity, biocompatibility, and sufficient mechanical properties to provide osteoblasts or direct osteogenesis and form a good material–bone tissue interface. Since it is difficult for a single-component bone repair material to simultaneously meet the above requirements, in recent years, research has mainly focused on the 3D printing of composite bone scaffolds for repairing bone defects. This technique can rapidly lay down continuous layers of material to create 3D solids using precise 3D stacking under computer control [23].

In this review, we begin by providing an overview of 3D printing technology for 3D-printed scaffolds in the context of repairing bone defects. Next, we delve into a comprehensive review of both single-component and composite scaffolds, produced through 3D printing. Finally, we conclude by highlighting the challenges encountered in current studies and offering recommendations for future research endeavors.

## 2. 3D Printing Technology for 3D-Printed Scaffolds

The 3D printing technology for bio-scaffolds can be divided into several key techniques: single 3D printing technology, multi-printing technology, multi-nozzle printing, and hybrid systems. Single 3D printing technology is simple to use, cost-effective, and suitable for printing bio-scaffolds that do not require multiple materials or complex structures. Currently, single 3D printing technologies widely used in tissue engineering include selective laser sintering (SLS), the stereolithography apparatus (SLA) process, fused deposition modeling (FDM), direct ink writing (DIW), direct energy deposition (DED) [24,25], and others [26,27]. The technologies for 3D printing composite materials mainly include inkjet, FDM, SLA, SLS, multi-printing technology, multi-nozzle device printing, hybrid systems, etc. [28]. 

SLS technology offers a fast processing speed and does not require the use of supporting materials. However, the surface of the printed product is rough and requires post-processing. Additionally, dust and toxic gases may be produced during the processing, and the continuous high temperature can cause polymer material degradation, deformation of bioactive molecules, or cell apoptosis [29]. SLA technology provides stable performance, high mechanical strength, high precision, and good surface quality. It can manufacture tissue engineering scaffolds with complex and intricate shapes. Its limitation is that it is only suitable for liquid photocuring resin materials, restricting the application of most biological materials. The FDM forming machine offers the advantages of a simple structure, no environmental pollution, no chemical change in the forming process, and small warpage deformation of the parts [30,31]. Nevertheless, the printing accuracy is limited, and the surface exhibits noticeable stripes. Moreover, the vertical direction strength is lower, necessitating the design and fabrication of support structures. DIW technology offers versatility in terms of ink types, including conductive elastomers or hydrogels. However, similar to FDM, it is constrained by the extrusion nozzle and has limitations regarding fiber diameter and accuracy. Multi-printing technology allows for the precise printing of different materials or cells at specific locations, enabling the fabrication of complex scaffold structures [32,33]. It offers advantages such as multi-material printing, high efficiency and speed, precise control, and high accuracy.

The multi-nozzle device enables the simultaneous deposition of multiple materials and cells, providing flexibility in regards to printing parameters and high efficiency [32,34]. However, it also exhibits challenges such as high equipment costs, requirements for rheological properties, and issues related to clogging and positioning [35]. The development of a multi-nozzle 3D bioprinting system for the fabrication of biological structures has been reported. This system can simultaneously print multiple types of cells and biological materials to construct complex tissue structures. Its advantages include high scalability, simultaneous deposition of multiple materials and cells, and increased printing speed. However, its disadvantages include increased complexity of the equipment and challenges in coordinating the movements of multiple nozzles. 

Hybrid systems combine multiple technologies to overcome the limitations of single techniques [36]. They enable more diverse and personalized designs for bio-scaffolds, allowing for the blending of multiple bio-materials and improved structural controllability [37]. Hybrid systems can generate innovative composite materials with better biological compatibility and mechanical performance. However, they also come with limitations such as complex equipment and difficulties in operation and debugging [38].

In the 3D printing process, most structures require the establishment of functions and working equipment in the post-printing process. The four main post-treatment steps are: (1) removal of the support structure and the implementation of secondary curing steps [39], (2) surface coating for functional and/or protective purposes [40], (3) improvement by polishing and eliminating surface roughness [41], and (4) modification of material properties and structural shapes [42,43]. Most 3D printing systems require the removal of support structures, as well as cleaning and secondary curing steps.

## 3. The 3D-Printed Single-Component Scaffolds 

In the past decades, various materials have been used to study the influence of scaffolds on osteogenesis and osseointegration, including metals [44], bioceramics [45], bioactive glasses [46], and biopolymers [47]. These scaffolds are mainly derived from natural and synthetic bioceramics, biopolymers, metallic biomaterial or their alloys, and composite biomaterials. Table 1 provides a comprehensive overview of the composition, mechanical and biological properties, 3D printing technology, advantages, and applications of different single-component scaffolds used for repairing bone defects through 3D printing.

### 3.1. Metallic Biomaterial Scaffolds

Metallic biomaterials mainly include stainless steel, cobalt-based alloys, and titanium and titanium alloys. Table 1 presents the mechanical and biological properties of metallic biomaterials and the advantages and disadvantages of their use for 3D printing. Stainless steel is the earliest and most successfully applied metal biomaterial. Although stainless steel is considered bioinert and biocompatible, it can induce tissue response, leading to osteonecrosis, foreign body granuloma, and acute and chronic inflammation [44]. Porous 316L stainless steel scaffolds were printed using the selective laser melting (SLM) technique, with 87% porosity, a 0.75 mm pore size, and a maximum compressive strength of 10.6 ± 0.6 MPa [48]. A high-power SLM laser printed 316L stainless steel scaffolds with good elongation and corrosion resistance. However, 316L scaffolds prepared at low power exhibited poor performance [49].

Cobalt-based alloys are used as medical implants because of their excellent mechanical properties and biocompatibility. Porous CoCr scaffolds were fabricated using SLM technology [50]. The printed porosity is 81.03%, the pore size is 0.625 mm, and the strength is 1279.52 MPa. The printed scaffolds exhibited a porosity of 81.03%, with a pore size of 0.625 mm and a strength of 1279.52 MPa. Furthermore, porous cobalt-chromium-molybdenum (CoCrMo) bone substitutes, with porosity, were 3D-printed using the electrophoretic deposition (EPD) technique. A Co-Cr-Mo scaffold with an 86% pore size was loaded with gentamicin and filamentous protein [51]. Nevertheless, the additive on the surface of the complex feature is ideal for knee implants due to its exceptionally high mechanical strengths, which are comparable to those of forged metal [24,52,53]. In 2019, Ryu et al. conducted in vitro and in vivo studies and found that DED technology with Ti-coated cobalt chromium (CoCr) alloys did not provoke chronic inflammatory reactions. This indicates that the technology exhibits biocompatibility.

However, the release of Co and Cr ions from this Co-Cr-Mo alloy scaffold is known to have cytotoxic effects. Additionally, the use of porous bone substitutes increases the risk of post-operative infection, mechanical mismatch, and biological neutrality. As a result, the application of cobalt-based alloys in vivo is limited [54].

On the other hand, titanium and titanium alloys have emerged as relatively new and widely used metal biomaterials [55]. Zhang et al. conducted a study in which they utilized selective laser melting (SLM) to fabricate titanium scaffolds with varying porosities and mechanical properties. These scaffolds were then transplanted into an animal model of Beagle dogs [56]. The scaffolds exhibited a porosity range of 66.1% to 79.5%, with a pore size of 0 ± 20 µm, and a compressive strength of 104.26 MPa. Beagle dogs implanted with these scaffolds showed no signs of inflammation, foreign body reaction, or any other adverse reactions at the implantation site.

Titanium possesses high strength and rigidity, and additionally, its surface oxide layer offers excellent corrosion resistance [57,58,59]. However, it is important to note that titanium and titanium alloy scaffolds have limited degradability. Consequently, they can release metal ions, which pose a potential risk to the human body after wear [60]. Despite the exceptional performance demonstrated by titanium alloys in medical repair procedures, they still release trace amounts of aluminum, vanadium, and nickel into the plasma during surface wear. This poses potential threats to the human skin, central nervous system, and kidney function [60]. 

The 3D printing of metal materials overcomes the adverse effects of high cost [61], complex processes [23], low material utilization [62], and difficulty in subsequent processing inherent in traditional manufacturing processes. However, when using this technology to form metal parts, the workpiece is prone to defects such as spheroidization, cracks, porosity, and warpage deformation due to the special processing properties of powder/wire or improper selection of process parameters [63,64,65]. These defects severely affect the mechanical properties of the metal and can lead to stress shielding after bone grafting. Moreover, the printing of metal scaffolds should be carried out under high-temperature conditions, and it is not possible to synchronize the coating of biologically active molecules or the mixed printing of cells during 3D printing [66]. As a result, future research will focus on modifying metal surfaces or adding other biomaterials to improve their performance for bone regeneration [24,67]. 

### 3.2. Ceramic Material Scaffolds

Bioceramic materials exhibit good bioactivity and can promote bone tissue regeneration and neovascularization [45]. Based on their activity, bioceramic materials can be classified as either bioinert and bioactive ceramics. Bioactive ceramics have more applications than inert ceramics due to their ability to chemically bond with tissues [68]. The customized 3D printing of ceramic materials for bioprostheses allows for the small batch manufacturing of complex components at low cost to meet patients’ needs for bone replacement. Some bioactive ceramics commonly used for 3D printing include hydroxyapatite (HA) [69] and β-tricalcium phosphate (β-TCP) [70]. 

HA is more stable than other calcium phosphate materials [71,72,73,74]. In the bone regeneration field, HA has been systematically used as a filling material for bone defects, an artificial bone graft, and a scaffolding material in prosthetic procedures [75]. HA is used in bone regeneration because it induces bone ingrowth and prevents osteolysis [76]. HA scaffolds can be obtained through many 3D printing methods. Among them, digital optical processing (DLP), which mixes HA with photosensitive materials and removes the organic material after molding using a photopolymerization reaction, is one of the effective methods for molding HA materials. In 2001, a resin mold prepared using DLP was developed for manufacturing HA scaffolds with designed internal structures [77]. The hole size of the HA scaffold was 366~968 μm, and the porosity was 26~52%. Porous HA scaffolds with engineered internal channels induced more new bone generation than porous HA scaffolds without channels [78]. Similarly, porous HA structures with interpenetrating pores were fabricated using light-cured processing molds for bone replacement [79]. Chengwei Feng cultured MC3T3-E1 cells for four weeks, which resulted in strong osteocalcin signal generation. HA scaffolds were directly fabricated with DLP, and porous HA bioceramics proved somewhat degradable [69]. In addition, in 2021, Yao et al. further explored the DLP printing of HA scaffolds with p-unit triple-cycle minimal surface structures [80]. After performing an in vitro culture of MSCs for seven days, scaffolds with p-cell structures exhibited higher cell density. The 3D-printed HA facilitated osteoblast adhesion and proliferation because of its laminar structure and its material rich in hydroxyl groups [81]. However, HA itself possesses insufficient fracture toughness and is susceptible to fatigue damage [82].

In conclusion, β-TCP exhibits excellent biocompatibility, biodegradability, and osteoinductivity. Since 1989, β-TCP has been used as a bone graft material for bone repair [83]. β-TCP has become a commonly used bone replacement biomaterial because of its good osteoinductive potential [84]. Furthermore, β-TCP implantation will not cause rejection, inflammation, or toxic side effects, making it conducive to the growth of new tissues [83,85]. In 2008, Vorndran et al. reported modifying a porous β-TCP scaffold with 5 wt% hydroxypropyl methylcellulose as the matrix material and a dry powder binder spray method to prepare porous β-TCP scaffolds, using water as the binder [86]. The final scaffolds prepared using this method exhibited low resolution, small specific surface area, and a maximum compressive strength of only 1.2 ± 0.2 Mpa, making their use as a repair material for bone defects difficult. In 2013, Tarafder et al. prepared porous β-TCP scaffolds using a binder spraying technique and investigated the effects of microwave sintering and pore size on the mechanical and biological properties of the scaffolds [87]. Microwave sintering enhanced the compressive strength by 46–69%. Scaffolds with a 0.5-mm pore size exhibited significantly higher cell density after culturing human embryonic osteoblasts (hFOB). The scaffold was implanted into male Sprague Dawley rats with femoral defects, and new bone generation was observed after two weeks. DLP technology, with high resolution, can also be used to print β-TCP material. Schmidleithner et al. evaluated the biological performance of DLP-printed β-TCP scaffolds [88] using the Mc3t3-e1 cell culture. The results showed a significant increase in alkaline phosphatase activity. However, one of the most significant drawbacks of β-TCP ceramic materials is their low mechanical strength. They cannot withstand large impact forces; therefore, they cannot be used as a replacement for load-bearing bones [89].

### 3.3. Bioactive Glass (BAG) Scaffolds

Bioactive glass is a ceramic material containing varying proportions of P_2_O_5_, SiO_2_, CaO, Na_2_O, Al_2_O_3,_ ZrO_2,_ and CaO. Some commercially available bioglass compositions include 45S5 bioglass, which binds to bone and soft tissues, and 5S4.3 bioglass (a high-calcium bioglass), which binds only to bone tissue. The main advantage of bioglass is its good bioactivity, and 45S5 is a widely used commercial bioglass for bone tissue engineering that facilitates osteogenesis, both in vitro and in vivo [90,91]. However, it is not suitable for load-bearing applications because of its poor mechanical properties.

A porous 45S5 glass material was fabricated using a direct 3D printing technique [90,91], with a porosity of 60.4%, a pore size of 1.001 ± 48 mm, and a compressive strength of 16.01 ± 1.53 MP, to simulate body fluid experiments. Culture experiments with human bone marrow stromal cells (hBMSCs) have shown that 45S5 scaffolds exhibit apatite mineralization capacity and good bioactivity. Lusquinos et al. [92] printed 45S5 and S520 bioglass scaffolds using selective laser sintering technology. Due to the lower melting point, printing bioglass materials using the powder fusion technology is much easier than it is with CaP ceramics. Tesavibul et al. [93] used 45S5 bioglass and acrylate-based photopolymer slurry, with a solid content of 43 wt%, to prepare a porous cell structure using light-curing technology. Baino et al. [94] prepared 45S5 bioactive glass and measured its compressive strength at 1.2 ± 0.2 MPa using the foam replication method, which was lower than the standard reference range of human trabecular bone of 2–12 MPa [95]. Kang et al. [96] prepared 45S5 bioglass by stereolithography and found that the maximum bending strength reached 37.9 ± 5 MPa when the volume fraction was 32–40 vol%. Although its mechanical properties have improved through 3D printing technology, BAG still faces the disadvantages of a high SiO_2_ content, a slow degradation rate, the mismatch with the rate of new bone formation, and the crystallization process of product particle components. Therefore, it is difficult to prepare scaffolds with a 3D network structure using the thermal sintering method for load-bearing bone repair.

Directional laser deposition (DLD), also known as laser engineered net shaping (LENS), is a laser-assisted direct additive manufacturing technology categorized under DED technology [97]. This technology is highly suitable for the manufacturing of BAG ceramic materials, offering several advantages such as a simple process, short production cycles, and low costs. These features make it particularly appealing for the fabrication of molten oxide ceramic materials. For instance, Balla et al. [98] successfully utilized the DLD method to produce crack-free alumina ceramics. The resulting samples exhibit anisotropy, which is determined by the honeycomb structure of aligned alumina along the deposition direction. Specifically, the compressive strength along the deposition direction measures 123 MPa, whereas the compressive strength perpendicular to the deposition direction is 229 MPa. Wu et al. [99], Li et al. [100], and Pappas et al. [101] employed this technique to investigate the impact of initial composition on Al_2_O_3_/ZrO_2_ melt-grown composites. Their research revealed that adjusting the composition ratio of zirconia effectively inhibits crack formation, optimizes the microstructure, and improves the mechanical properties.

### 3.4. Polymer Scaffolds

Commonly used biopolymer materials in 3D-printed bone composite scaffolds include natural and synthetic polymers [47]. Naturally degradable polymer materials mainly include collagen [102,103], gelatin [104], chitosan [105], alginate, hyaluronic acid, etc. The advantages of this material are non-toxicity, good hydrophilicity, and excellent biocompatibility [47]. In the bone regeneration field, collagen [106], gelatin [107], and chitosan [105] are more researched and applied. 

Collagen, one of the most commonly used scaffold materials in bone tissue engineering, exhibits good degradability, but its mechanical properties are poor, its osteoinductivity is poor, and it is not easy to manipulate during surgery [102,103]. Gelatin is a product of the partial degradation of collagen; it has good biocompatibility and degradability, shows no adverse effects on humans, is widely available, and is inexpensive. Gelatin molecules contain a variety of functional groups with high reactivity, and they can be easily modified and cross-linked for specific functions [104]. In addition, Gelatin can be dissolved in hot water above 45 °C. After cooling, the gelation of gelatin occurs. Because of these characteristics, gelatin is widely used in the bone regeneration field. Choi et al. [108] used 3D printing technology to prepare a gelatin scaffold with aperture diameters of 400–800 μm, the mechanical properties of which increased with increased gelatin solution concentration. Experiments showed that the cells adhered to and proliferated well in the gelatin scaffold. Dong et al. investigated the effect of different pore sizes on the proliferation of osteoblastic fibroblasts on gelatin scaffolds [108], reporting that fibroblast growth could be accelerated when the pore size was >580 μm and the porosity was >83%. As a scaffold material, gelatin exhibits the main disadvantages of poorer mechanical properties and a faster degradation rate. Cross-linking, chemical grafting, and blending with synthetic polymer materials in regards to gelatin materials can further improve the mechanical properties of the gelatin scaffold and prolong the degradation time. Chitosan, which is the product of the deacetylation of chitin, is an essential natural biomaterial. Chitosan possesses good antibacterial properties, hydrophilicity, biodegradability, and biocompatibility; thus, it can improve the adhesion, proliferation, and differentiation of osteoblasts [109]. Therefore, chitosan is widely used as a bone tissue engineering scaffold. The main disadvantages of chitosan are its solubility in only acidic aqueous solutions, along with its poor strength, and its toughness.

The main synthetic polymers include polylactic acid (PLA), polyglycolic acid (PGA), and copolymers of both (Poly(D,L-lactic-co-glycolic acid), PLGA), along with polycaprolactone (PCL), polyhydroxy butyrate (PHB), etc. [110]. Among them, PLA is the most commonly used, belonging to the category of α-polyester, which is an important biomaterial. It is non-toxic, non-irritating, and easily processed. The final degradation products are CO_2_ and H_2_O, which can be metabolized or excreted by the human body through normal pathways [111]. The levorotatory isomer of PLA is called levorotatory polylactic acid (PLLA), which possesses good mechanical properties. Its degradation product, levulinic acid, can be absorbed by the body through metabolism. PLLA is mainly used in bone tissue engineering scaffolds [112,113,114]. Ju et al. utilized supercritical carbon dioxide (Sc-CO_2_) foaming technology to prepare the PLLA scaffold. Based on a previous experiment, its porosity was measured at 90.3%, and its mechanical strength was 11.9 MPa [115]. However, when used for the preparation of bone tissue engineering scaffolds, the PLLA scaffold has certain performance deficiencies. The three main aspects are as follows: (1) PLLA is a hydrophobic polyester,, with poor hydrophilicity. Therefore, it is difficult for the cell culture fluid to fully wet the surface of the scaffold’s pore wall, which affects the adhesion and proliferation of cells on the scaffold surface. (2) Although PLLA is degradable, its degradation rate is slow, and the accumulation of its degradation product, lactic acid, in the body can easily lead to complications such as inflammation and swelling. (3) PLLA is a linear structural polymer, with poor heat resistance and toughness, a lack of flexibility and elasticity, and insufficient mechanical strength [116,117,118].

PLA (polylactic acid) is a commonly used material in 3D printing [119]. It is frequently utilized for applications in bone fixation repair and tissue engineering scaffolds [120,121,122,123,124,125,126]. PLA’s high thermoplasticity and biodegradability make it an excellent choice for such purposes. It is worth noting that PLA has been approved by the US Food and Drug Administration (FDA) for implantation in humans [127]. In a specific study, preheated 3D-printed structures were shaped into porous cylindrical scaffolds to promote the osteogenesis and mineralization of human fetal osteoblasts (hFOB) [128]. The porous scaffold had a porosity of 69.3 ± 7.4% and a pore size of 1 ± 0.1 mm. Its maximum compressive stress was measured at 41.38 MPa. After 28 days of hFOB culture, significant osteogenic differentiation was observed in the hFOB cells.

PCL (polycaprolactone) is a biodegradable polyester known for its non-toxicity and histocompatibility [129]. It can withstand harsh physical and chemical conditions due to its adjustable chemical, biological, and mechanical properties [130]. Mehraein et al. studied the mechanical properties of PCL scaffolds under different fused deposition manufacturing (FDM) process parameters and reported a tensile strength of 16.086 ± 0.247 MPa under optimal conditions [131]. Williams et al. designed six cylindrical porous PCL scaffolds using selective laser sintering (SLS) [132]. Primary human gingival fibroblasts (HGF) were seeded on the scaffolds and implanted into immunodeficient mice, aged 5–8 weeks. After 4 weeks, a significant amount of bone growth was observed on the external surface of the scaffold and inside the scaffold holes. The slow degradation rate of PCL has been acknowledged by the US FDA, resulting in its approval for tissue engineering applications in the human body [133,134]. Therefore, PCL scaffolds play an active role in bone repair and regeneration.

### 3.5. Nanofiber Scaffolds

Nanocellulose materials as natural polymers, exhibiting high strength, high specific surface area, high hydrophilicity [135], biodegradability, histocompatibility, and low toxicity, have stimulated extensive research interest in the biomedical field in recent years, especially in regards to bone tissue engineering. Incorporating nanocellulose into scaffolds can enhance biocompatibility and facilitate cell adhesion and proliferation [136]. Moreover, the incorporation of nanocellulose enhances the crystallinity of the composite scaffold and slows down the depolymerization rate, decreasing the overall degradation rate of the scaffold [137,138].

The materials used to produce nanofibers include natural macromolecules and synthetic polymers. The natural macromolecules include collagen, hyaluronic acid, gelatin, chitosan, elastin, etc. [139]. Synthetic polymers mainly include polylactic acid (PLA), polyglycolic acid (PGA), PCL, polylactic acid-hydroxyacetic acid copolymer (PLGA) and its copolymers, and polyurethane (PU).

Among them, hydroxybutyric acid and hydroxyvaleric acid (PHBV) have attracted increasing attention due to their good biodegradability, non-immunogenicity, and biocompatibility. PHBV is a copolymer of polyhydroxy butyric acid (PHB) and polyhydroxyvaleric acid (PHV). Sevastianov et al. [140] reported that PHBV coming in contact with blood did not affect the hemostasis system at the cell response level. Furthermore, cells mounted no inflammatory response to the material. In addition, fibroblasts, endothelial cells, and hepatic parenchymal cells cultured on the PHBV membrane showed good cell adhesion. The study also proved that PHBV can be used as an extracellular matrix to design simulations [141].

**Figure 1:**
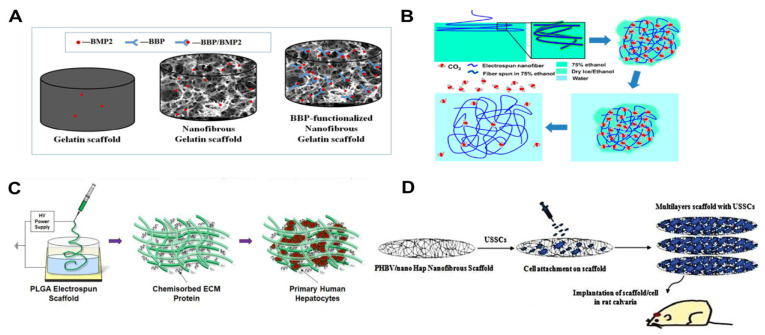
Nanofiber scaffolds for a bone defect. (**A**). BBP functionalized gelatin scaffold [142]. (**B**). A 3D PLA nanofibrous scaffold obtained using a modified CO_2_ foaming technique [145]. (**C**). PLGA wet electrospun nanofiber scaffold [143]. (**D**). Nanofibrous PHBV/nano-HAp scaffolds filled with USSCs [144].

Yao et al. combined thermally induced phase separation and porogen leaching technology (TIPS&P) to prepare gelatin nanofiber scaffolds. These functional scaffolds were cross-linked to BMP-binding peptides (Figure 1A) [142]. BBP modification greatly enhanced the BMP2 binding and retention capacity of the nanofibrous scaffolds without affecting their macro/microstructure and mechanical properties. Importantly, BBP-functionalized gelatin scaffolds were able to significantly promote BMP2-induced osteogenic differentiation. Jing et al. developed a 3D PLA scaffold using electrospinning technology combined with CO_2_ overflow foaming technology (Figure 1B) [142]. This 3D scaffold’s continuous nanofiber structure provides a large specific surface area and porosity, allowing cells to display higher cell viability and faster proliferation rates. Similarly, Brown et al. used a newly optimized wet electrostatic spinning technique to prepare type I collagen-modified nanofibrous PLGA scaffolds (Figure 1C) to mimic the in vivo extracellular matrix (ECM) structure. Electrostatically spun composite nanofibers of PHBV and HA can better mimic the micro/nanostructure of natural bone [143]. Biazar et al. used an electrostatic spinning device to prepare PHBV/nano-Ha/USSCs nanofiber scaffolds (Figure 1D), which were implanted into the cranial defect site of male Wistar rats, significantly increasing the ossification rate [144].

**Table 1:** The 3D printed single-material scaffolds.

Category	Material	Material Mechanical Strength (MP)	Scaffold Mechanical Characteristics			Biological Performance			The 3D Printing Technology	Advantages	Disadvantages	Application	Ref.
			Hole Size (mm)	Porosity(%)	Strength (MP)	Degradability	Osteoinductivity	Biocompatibility					
Me tallic biomaterial	Ti-6Al-4V alloy	Ultimate tensile strength: 240–860	-	-	Ultimate tensile strength: 860	Corrosion resistance	Stimulating the growth of bone tissue in the weight-bearing area	Low toxicity	-	Low shear strength and low wear resistance	A possible toxic effect resulting from released vanadium and aluminum	Orthopedic and dental implants	[55]
	Ti-6Al-4V alloy	Ultimate tensile strength: 240–860	0.65 ± 0.02	66.1–79.5	Compressive strength: 36.45–140.26	Difficult degradation, surface corrosion resistance	Stimulating the growth of bone tissue in the weight-bearing area	No inflammation in vivo	SLM	Personalized customization, low processing cost	Surface wear releases aluminum, vanadium, and nickel, which are toxic	The transplantation of a Beagle’s right posterior femoral head	[56]
	316Lstainless steel	Ultimate compressive strength:c981	0.75	87	Compressive strength: 10.6 ± 0.6	Difficult degradation	-	Negative effect on interactions with cells	SLM	No negative influence on material biocompatibility	Negative effect on interactions with cells	Tool for bone defects repair	[48]
	316Lstainless steel	Yield strength: 299–295	-	0.05–1.31	Yield strength: 470–480	-	-	Poor immersion	SLM	Reasonable cost, sufficient corrosion and fatigue resistance, and ease of welding and fabrication	Poor immersion, prone to instability and fracture	Surgical implants	[49]
	Co-Cr-Mo	Compressive strength: 600–800	0.625–0.875	60–82	Compressive strength: 271.53–1279.52	High corrosion resistance, difficult degradation	Cell Proliferation	Supporting cell adhesion	SLM	Personalized customization	-	Model use for cortical and trabecular bones	[50]
	Co-Cr-Mo	Compressive strength: 600–800	0.625 ±0.054	-	Tensile strength 3.43 ± 0.38	-	The higher the laser power, the better the bone cell growth	Harmful effects of cobalt and chromium ions on osteoblast production	EPD	Good cell spreading, proliferation, and cytotoxicity	The released Co and Cr ions have cytotoxic effects	Coated gentamicin-loaded silk fibroin	[51]
Ceramic material	HA material	Compressive strength: 462–509	1.15–1.21	49.32–54.52	Compressive strength: 9.3–21.4	Excellent degradation	-	-	DLP	Good mechanical properties and biocompatibility	Lack of in vivo and in vitro tests	Bone tissue engineering	[69]
	HA material	Compressive strength: 462–509	-	74%	Compressive strength: 4.09	-	-	Non toxicity to rBMSC cells	DLP	High repeatability and accuracy.	Poor mechanical properties, high brittleness	rBMSCs cultured in vitro	[80]
	β-TCP material	Compressive strength: 5.1–10.87	0.5–1	27–41	Compressive strength: 6.62–10.95	Fast degradation	Both micropores and macropores promote osteogenesis in a rat model	Significantly high density of living cells	Direct 3DP	Similar to bone mineral composition. the scaffold is made directly from CaP. powder.	Poor mechanical properties	Femoral defect model of the Sprague Dawley rat	[87]
	45S5 bioglass	Compressive strength: 500			The flexural strength: 37.9 ± 5	High content of SiO_2_, the rate of degradation does not match the rate of new bone formation	The surface of MBG has newly formed apatite, with excellent biocompatibility	-	Direct ink writing	The 3D printed MBG scaffold has apatite mineralization ability and long-lasting drug delivery properties	High brittleness, crystallization trend	hBMSCs cultured in vitro	[96]
	Mesoporous bioactive glass	Compressive strength: 0.06	0.624 ± 0.04	60.4%	Compressive strength: 16 MPa	Fast degradation	-	Excellent apatite mineralization ability	Direct 3DP	Excellent biocompatibility	Uncontrollable pore architecture, low strength, high brittleness, and the requirement for a second sintering	Excellent candidate for bone regeneration	[46,96]
Polymer Materials	Gelatin	Compressive strength: 0.92	0.20	75	Compression modulus: 0.38	Fast degradation	Promoting chondrocyte differentiation	Excellent bio-compatibility	DLP	Excellent biocompatibility, desirable osteoinductivity	Poor mechanical properties, fast degradation	hADSC cultured in vitro	[104,108,146]
	Gelatin	Compressive strength: 0.92	0.436–0.777	>82	0.0090–0.0418	Fast degradation	-	HDFs cell proliferation	Low-temperature freezing system	Controlled porosity	Poor mechanical properties, gelatin has a poor printability	HDFs cultured in vitro	[104,108,146]
	Gelatin	Compressive strength: 0.92	-	70–75	Compressive strength: 0.023–0.115	Fast degradation	Accelerate bone regeneration, maintaining a stable mechanical environment	hADSC cell proliferation	Extrusion-based low-temperature 3D printing	Promotes articular cartilage regeneration	Poor mechanical properties	hADSC cultured in vitro	[104,108,146]
	Poly (L) Lactic Acid	Strength: 32.79	-	-	Strength at break point: 25.43–29.5	Good degradation property	-	Promoting adhesion of human fibroblasts	-	Good wettability characteristics, biocompatibility and biodegradability in a pH value	Poor hydrophilicity, poor mechanical properties	Antitumor therapy, gene transfer agents, targeted drug delivery systems, light harvesting materials	[117,128,147]
	Polylactic acid	Compressive strength: 76.1	-	3.4–69.3%	Spiral of 41.38, porous spiral of 29.13, porous cylinder of 16.04	Slow degradation rate	Promoting hFOB cell adhesion, proliferation, and mineralization	High activity of hFOB cells	Direct 3DP	Provide complex structures and growth areas to capture and induce cell ingrowth	Weak cell affinity	hFOB cultured in vitro	[117,128,147]
	Polycaprolactone	Ultimate tensile strength: 24	-	-	Tensile strength: 16.086 ± 0.247	Hydrophobicity, slow degradation	-	-	FDM	Geometric flexibility in the design	-	-	[129,131,148]
	Polycaprolactone	Compressive strength: 11.9	0.515	70–80	Compressive strength: 6.38 ± 0.82	Slow degradation	Guiding bone regeneration through its honeycomb-like microarchitecture	Inducing recruitment of natural bone progenitor cells and promoting cell retention	-	Accelerate healing of the segmental defect	Poor mechanical properties	Rabbit ulna transplantation experiment	[129,131,148]

## 4. The Composite Scaffolds for Repairing Bone Defects

Bone tissue engineering composite scaffolds are produced by combining two or more biomaterials in a specific ratio to synthesize the advantages of various materials, offering biocompatibility, mechanical strength, and a bionic structure similar to natural bone [28]. In addition, 3D-printed composite scaffolds can control the scaffold structure and scaffold pore structure and continuously improve the internal connectivity performance. Bone repair composite scaffolds are generally divided into two categories: bioceramic bone cement composite scaffolds and polymeric composite scaffolds. Table 2 presents a comprehensive summary of the composition, 3D printing technology and printing equipment utilized, advantages, and applications of diverse composite scaffolds fabricated through 3D printing for the purpose of repairing bone defects.

### 4.1. Bioceramic Bone Cement Composite Scaffolds

There are many types of bone cement materials, including calcium sulfate (CSC), CPC, and polymethyl methacrylate (PMMA), each of which have undergone many basic research and clinical applications [149,150]. In addition, several bone cements with different characteristics have been mixed to prepare composite bone cements, such as PMMA and β-TCP [151], PMMA and HA [152], PMMA and CPC [153], etc. Currently, CPC and PMMA injectable bone cement have many clinical applications [154].

#### 4.1.1. PMMA Bone Cement Scaffolds

PMMA bone cements are characterized by high compressive strength, workability, biocompatibility, and visualization; they can effectively relieve patients’ pain and stabilize vertebral fracture ends [155,156,157]. However, PMMA bone cement has some shortcomings, such as non-biological activity, non-absorbability, MMA monomer toxicity, a high setting temperature caused by the exothermic reaction, monomer leakage, etc. [158,159]. In particular, PMMA bone cement polymerization can create microgaps at the bone–cement interface due to volume shrinkage [160,161,162], leading to aseptic loosening or reduced local mechanical strength. This aseptic loosening of the bone–cement interface has attracted increasing attention in recent years [163]. Secondly, PMMA bone cement has an excessive elastic modulus [164], easily leading to secondary fractures in the adjacent vertebrae. Therefore, PMMA is rarely used as a separate material to create bone repair scaffolds. Currently, for repairing bone, PMMA can only be used as a filler material in percutaneous vertebroplasty (PVP) and percutaneous kyphoplasty (PKP) after repairing osteoporotic vertebral compression fractures.

To compensate for the shortcomings of PMMA, some researchers have focused on modifying and improving PMMA bone cement. Mixing PMMA with other biological materials to increase porosity is an effective method for achieving this goal. Deering et al. coated PMMA-Al_2_O_3_ layers on porous stainless steel implants printed using SLM technology [165], effectively enhancing the internal pores of the scaffolds. The Saos-2 osteosarcoma cells adhering to the coating exhibited high activity. In addition, bone cements with good mechanical strength were obtained using relatively low-strength calcium phosphate bone cement compounded with PMMA bone cement [166]. De Santis et al. prepared copper-doped tricalcium phosphate (Cu-TCP) particles combined with PCL/PMMA scaffolds to repair cranial defects [167]. The introduction of Cu-TCP enhanced the mechanical properties of the scaffolds. However, this method was only analyzed by virtual and physical models, and no relevant cellular and animal experiments were performed. In addition, Esmi et al. prepared PMMA/CNT/HAp nanocomposites using the FDM technique. By 3D printing this HAp nanocomposite, good interlayer adhesion was achieved. The introduction of CNT particles could improve the viability and growth of L929 mouse fibroblasts.

#### 4.1.2. CPC Bone Cement Scaffolds

CPC is a new type of bone defect repair material developed by Brown and Chow in 1985 [168]. CPC has many advantages over conventional sintered ceramics. CPC forms weak crystalline HA through a hydration reaction and can easily simulate osteoporosis by adding trace elements, thus showing excellent biocompatibility and bone conductivity. CPC also exhibits good plasticity and can fill complex bone defects. Moreover, the weakly crystalline HA formed by the hydration reaction of CPC has better degradability than the sintered HA. However, CPC still has some disadvantages, such as poor mechanical properties and slow degradation. The strength of CPC can only reach the strength of cancellous bone, which differs significantly from that of dense bone. Moreover, CPC is a brittle material with low fracture resistance and reliability, which limits its application in load-bearing areas. Since the pores in CPC-cured bodies are mostly in the submicron and nanoscale ranges, the lack of connected macropores above 100 μm is not conducive to its growth into the material’s interior and the early formation of blood vessels, resulting in the slow degradation of CPC.

**Figure 2:**
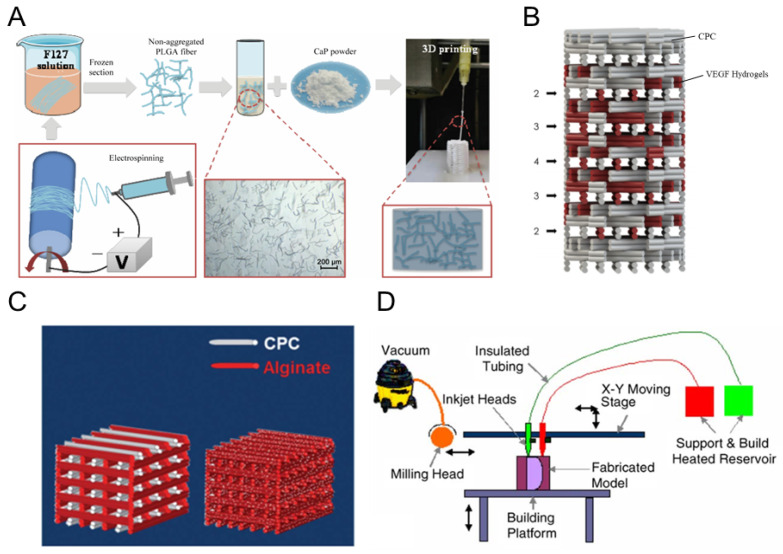
The 3D-printed CPC bone cement composite scaffolds. (**A**). The 3D printing process of CaP slurry containing PLGA fibers [173]. (**B**). the biphasic CPC-alginate scaffold (left) and the hybrid CaP-alginate scaffold (right) [177]. (**C**). Scanning electron microscopy of the PCL/CaP composite scaffold with a pore size of 600 μm [175]. (**D**). Model of the composite scaffold loaded with VEGF and CPC [176].

In order to overcome the disadvantages of slow degradation and poor toughness of CPC, studies have been undertaken to incorporate PLGA microspheres and fibers into CPC [169,170,171]. For example, in 2013, Hoekstra et al. prepared bone substitutes with PLGA microspheres loaded with calcium phosphate [172]. However, due to the degradation of the PLGA microspheres, the fibers could not generate three-dimensionally connected macropores in CPC. The use of 3D printing technology enables the preparation of 3D polymer networks and the precise control of the shape and size of pores, fiber size, connectivity, and porosity in the polymer networks. In 2020, an electrospun PLGA fiber/CPC scaffold was fabricated by extrusion-based 3D printing technology (Figure 2A), which significantly improved the toughness, biocompatibility, and osteogenic differentiation of the CaP scaffold [173]. In addition, one study incorporated wollastonite (WS) and CPC into PLGA scaffolds prepared through 3D printing. This approach was effective in enhancing the scaffold degradation properties and flexibility. Moreover, the adhesion and proliferation of mBMSCs were significantly improved because of incorporating WS [174]. Furthermore, CPC can be combined with organic materials to improve its properties. In 2013, Luo et al. printed a biphasic CaP/alginate composite scaffold using multichannel 3D plotting (Figure 2B). The high concentration of sodium alginate improved the scaffold’s mechanical strength and toughness. hMSCs cell culture experiments confirmed the cytocompatibility of the scaffold [175]. Polymer compounds have also been combined with CPC to form composite scaffolds. Mondrinos et al. printed PCL/CaP scaffolds using the drop-on-demand (DDP) 3D printing system, with an interconnected porous structure and a pore size of up to 600 μm for PCL/CaP composite scaffolds (Figure 2D) [176]. Human embryonic pallial mesenchymal stem cells (HEPM) were cultured on the PCL/CaP scaffold, and the number of cells increased after five days.

Incorporating growth factors into the CPC scaffold enhanced osteoinductivity. A study used 3D plotting to prepare CPC-bound vascular endothelial growth factor (VEGF)-rich alginate hydrogels for bone scaffolds (Figure 2C), resulting in a higher strain at break compared to that of pure CPC scaffolds [177]. In addition, incorporating VEGF into CPC during scaffold printing significantly increased the proliferation rate of endothelial cells. Similarly, incorporating bone morphogenetic protein-2 (BMP-2) could accelerate bone reconstruction [178]. Fenelon et al. prepared CPC/BMP-2 scaffolds using 3D bioprinting and human amniotic membrane using a two-step Masquelet’s induced membrane (MIM) technique. Li et al. prepared RhBMP-2/mesoporous silica (MS)/CPC scaffolds [179]. The incorporation of the appropriate MS facilitated the mediation of scaffold angiogenesis and osteogenesis. hBMSCs cells in culture and a male New Zealand rabbit bone defect model confirmed the biocompatibility and the osteogenic nature of the scaffold.

### 4.2. Polymer Composite Scaffolds 

For many years, attempts have been made to mimic natural bone by preparing inorganic–organic complexes. Organic polymeric materials are usually used to prepare the organic components of inorganic–organic complexes [180]. The organic polymeric materials used to prepare organic compounds usually include chitosan, gelatin, polylactic acid (PLA), poly(lactide) (PCL), and poly (lactide-polyhydroxyacetic acid) (PLGA).

#### 4.2.1. Chitosan Hydrogel Composite Scaffolds

**Figure 3:**
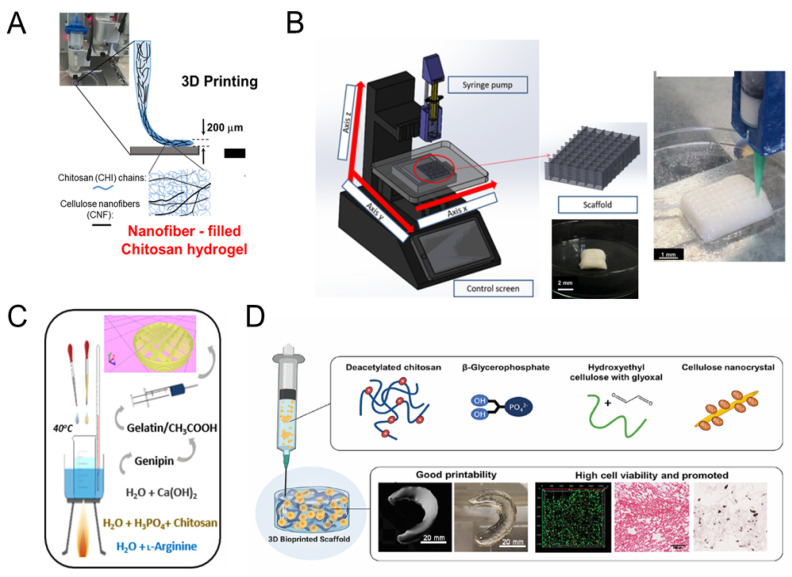
The 3D-printed chitosan composite scaffolds. (**A**). The 3D hydrogel structure of chitosan/cellulose nanofibers [195]. (**B**). The device used for the 3D-printed HA/Chitosan/PVA scaffolds [193]. (**C**). The 3D-printed HA/chitosan/genipin composite scaffolds [188]. (**D**). the extrusion-bioprinted CNCs/chitosan bio-ink for bone defect scaffolds [196].

Chitosan is a natural polysaccharide polymer obtained by the deacetylation of chitin. Chitosan has unique physical, chemical, and biological properties, including cationic polyelectrolytes, multifunctional base reactivity, good biocompatibility, etc., due to the amino groups in the molecular chain. In addition, the monomer has many hydroxyl groups, resulting in good biocompatibility and biodegradability. Chitosan is degradable in the body, and the body can absorb its degraded low-molecular-weight glucose without immunogenicity. Meanwhile, chitosan exhibits a variety of biological activities, such as blood coagulation, antibacterial activity, anti-tumor activity, immune regulation function, etc. [181]. However, chitosan has low solubility in water and other organic solvents, limiting its application in tissue repair and reconstruction [182,183]. In addition, chitosan is prone to deformation due to its poor mechanical properties and high swelling rate. To compensate for its shortcomings, chitosan is often mixed with other materials such as alginate [184], gelatin [185], sericin protein [186], and hydroxyapatite during the preparation of chitosan scaffolds. Each of these retains osteogenic activity and provides good mechanical properties for the composite. One study used 3D printing for composite graphene, gelatin, chitosan, and tricalcium phosphate. The compressive strength and Young’s modulus of the scaffold improved significantly [187]. Zafeiris et al. printed hydroxyapatite–chitosan–kynylpin scaffolds using the direct ink writing (DIW) technique. This study combined 3D printing with a lyophilization process (Figure 3C) to ensure the formation of independent structures and simulated the nanoscale porosity and interconnectivity of bone [188].

Hydrogels have a hydrophilic network structure [189]. Incorporating chitosan into this structure can mimic the extracellular matrix (ECM) and provide a microenvironment for cell proliferation [190,191]. Combining PHBV/calcium sulfate hemihydrate (CaSH) scaffolds with chitosan hydrogels has been reported [192]. CaSH could increase the scaffold’s compressive strength up to 16 MPa at 20% CaSH, while incorporating PHBV promoted the growth and adhesion of rat bone marrow stromal cells (rBMSCs). Another study used 3D printing to fabricate a hydroxyapatite/chitosan/polyvinyl alcohol (PVA)-based scaffold, which exhibited an elastic modulus similar to that of natural bone. The scaffold showed good biocompatibility and an enhanced attachment and proliferation of MSCs [193] after being loaded with BMP-2 (Figure 3B).

Nanofibrous materials are widely used in bone tissue engineering because their physical properties are similar to those of ECM [194]. Nanofibrous materials can be added to chitosan scaffolds. Tamo et al. used micro-extrusion (EBB) technology to print a chitosan hydrogel filled with cellulose nanofibers (Figure 3A). Natural cellulose nanofibers provided unique mechanical properties for the composite hydrogel, ensuring good printing ability and resolution of the printing structure [195]. Cellulose nanofiber-filled chitosan hydrogels contain more living NIH/3T3 fibroblasts than individually prepared chitosan scaffolds. Maturavongsadit et al. fabricated scaffolds based on 3D extrusion printing technology using nanocellulose (CNCs)/chitosan-based bio-ink (Figure 3D). CNCs significantly improved the viscosity of the bio-ink and the mechanical properties of the chitosan scaffold [196]. Also, the presence of CNCs promoted the osteogenesis of MC3T3-E1 cells in chitosan scaffolds, as evidenced by the upregulation of alkaline phosphatase activity, calcium mineralization, and extracellular matrix formation.

#### 4.2.2. Gelatin Composite Scaffolds

Gelatin is a partially degraded product of collagen which is widely used in bone repair. It is biodegradable and can be used as a scaffold material for regenerative bone tissue regeneration. Using gelatin as a scaffold for bone tissue repair offers the advantages of good water solubility, low immunogenicity, good histocompatibility, and low price [197]. However, gelatin materials have significant limitations, such as easy degradation and poor mechanical properties. Gelatin methacrylate (GelMA) is often used to improve various properties of gelatin. GelMA is derived from denatured collagen, which consists of methacrylamide and methacrylate groups [198]. Under UV light, it can crosslink to form hydrogels with various adjustable mechanical and physicochemical properties. However, GelMA, with its photoinitiated crosslinking characteristics, also presents significant challenges as a printing ink [199]. GelMA cannot be successfully extruded at concentrations below 7%, and high-concentration printing limits cell hybrid printing; it is also prone to clogging and unsmooth, cracked scaffold fiber lines. Therefore, other polymers should be used in combination with GelMA to improve printing performance.

Often, nHA, bioglass, and medical polymers such as PCL, PLA, and PEG are combined with gelatin [200,201,202]. Ye et al. incorporated bioglass into gelatin/sodium alginate, improving the mechanical strength and surface mineralization of the composite scaffold [203]. The Si and Ca^2+^ released from the bioglass facilitated the adhesion and proliferation of cells. The scaffold significantly promoted the proliferation and differentiation of mBMSCs cells. An increase in CNCS similarly enhanced the composite scaffold’s compressive strength and surface wettability [204]. Liu et al. further prepared a three-layer gradient GelMA/nHA scaffold [205]. The results showed that with a three-layer scaffold, the new tissue in the defect was better integrated with the surrounding tissue, the articular surface of the defect was smoother, and an increased amount of cartilage-specific extracellular matrix and type II collagen were observed.

#### 4.2.3. PLA Composite Scaffolds

Monolithic PLA scaffolds are brittle and have poor ductility, show slow degradation rates, and are not easily surface-modified. Concerning these drawbacks, the two main strategies to improve the performance of PLA are chemical modification and physical blending [206]. For chemical modification, a large amount of organic solvent is usually required, the reaction process is not easily controlled, and the production efficiency is low. Concerning physical blending, ceramics such as HAp or other biodegradable polymer materials, such as poly(lactone) (PCL) [207] and poly(ethylene glycol) (PEG) [208], are usually used, which are then blended with PLA.

**Figure 4:**
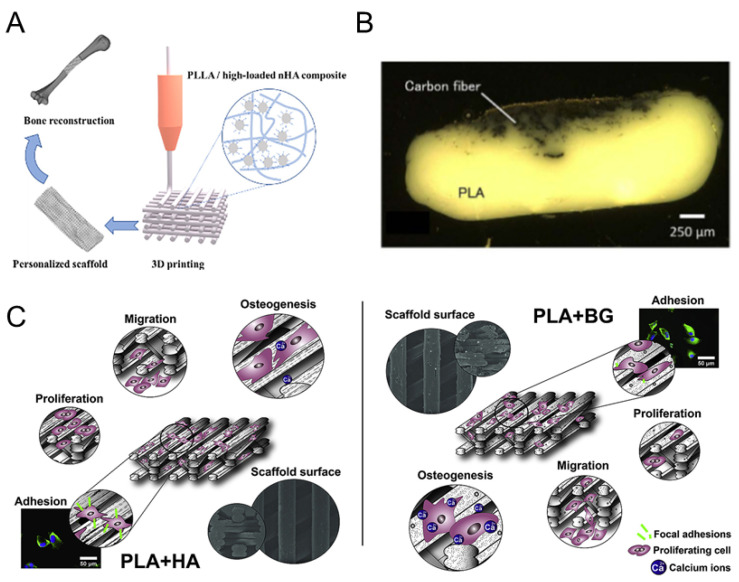
The 3D-printed PLA composite scaffolds. (**A**). The FDM-printed PLLA/nHA composite scaffold [212], (**B**). the FDM-printed cross-section of fiber-reinforced thermoplastic composites (FRTPs) [216], (**C**). a comparison of differentiation process induced by FDM-printed PLA + HA and PLA + BG scaffolds [213].

PLA is most commonly used as an HA/synthetic polymer composite [209,210]. In early studies on HA/PLA composites, PLA acted as an excipient, enabling HA particles to be compounded with it and processed into a rigid material of the desired shape. The reported tensile strength of high molecular weight PLA complexes with the inclusion of HA is about 10–30 Mpa, the flexural strength is about 44–280 Mpa, the compressive strength is about 78–137 MPa, and the Young’s modulus reaches 5–12 GPa [211]. Zhang et al. successfully prepared PLLA/nHA porous bone repair scaffolds using the FDM technique (Figure 4A), reporting that the compressive strength of the composite decreased from 45 to 15 MPa as the concentration of nHA in the composite increased from 0% to 50%. The higher the nHA content, the faster the loss rate of the scaffold. During degradation, CaP whiskers were found on the surface of 50% nHA scaffolds, demonstrating their good bioactivity [212]. Alksne et al. compared the effect of 3D-printed porous PLA + HA (10%) and the PLA + BG (10%) composites on rat dental pulp stem cells in vitro (Figure 4C). The PLA/BG composite scaffolds exhibited better biocompatibility and osteoinductive properties than did the pure PLA and PLA/HA scaffolds [213].

PCL exhibits good toughness and thermal properties, and its thermal stability is higher than that of PLA. Chen et al. prepared a PLA/PCL/TiO_2_ composite scaffold using FDM printing to enhance the mechanical properties of PCL [214]. Incorporating TiO_2_ improved the composite scaffold’s stability, tensile strength, and fracture strain. After culture experiments with MC3T3-E1 preosteoblasts, good cell adhesion on the composite surface was demonstrated. Li et al. prepared a PLA/PEG/nHA/dexamethasone composite scaffold using 3D printing technology [208]. Although incorporating Dex was detrimental to the proliferation and development of MC3T3-E1 cells in the early stage, it could promote osteogenic differentiation and in vitro mineralization in the later stage [215].

Loading small amounts of BMP-2 in PLA scaffolds can significantly affect the amount and repair of regenerated bone. Bouyer et al. designed a bionic polyelectrolyte membrane scaffold made of FDM3 coated with loaded BMP-2. High doses of BMP-2 released from the polyelectrolyte membrane resulted in the formation of well-mineralized and vascularized bone. Preparing 3D-printed polymer composites with high mechanical properties and excellent functionality by adding particle or fiber fillers to polymers is also a promising approach. Matsuzaki et al. printed continuous carbon fiber-reinforced PLA composites using a 3D printing method based on a fused deposition model (Figure 4B), which resulted in a tensile strength and modulus of 185.2 MPa and 19.5 GPa, respectively [216].

#### 4.2.4. Poly(lactide) (PCL) Composite Scaffolds

**Figure 5:**
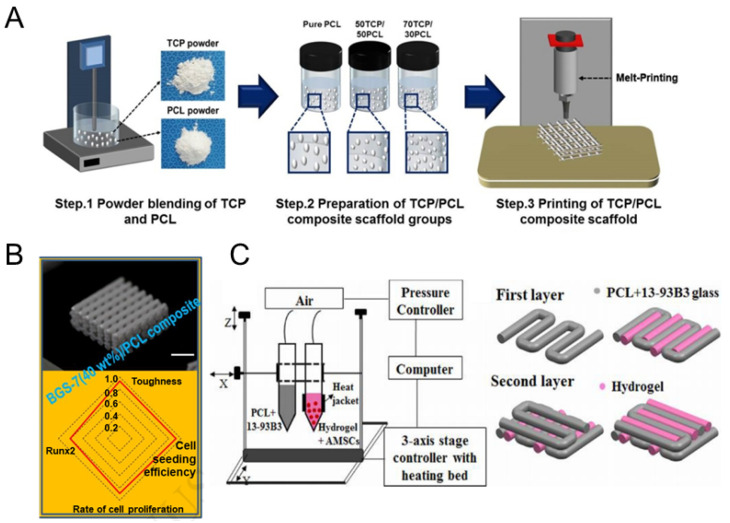
The 3D-printed PCL composite scaffolds. (**A**). The TCP/PCL composite scaffolds with different TCP and PCL content [220]. (**B**). Optical images of the PBGS-40 scaffold and its radial graphs demonstrating normalized toughness and in vitro cellular responses [222]. (**C**). The 3D printing of the PCL + 13-93B3 glass composite and hydrogel [223].

Polycaprolactone (PCL) is a synthetic polymeric organic compound with good biocompatibility and biodegradability, suitable for use as the main material in bone tissue engineering scaffolds. However, it is not biologically active, has a smooth surface, is hydrophobic, is not suitable for osteoblast adhesion and bone tissue regeneration, has poor mechanical strength, and degrades too slowly. Therefore, the modification and optimization of PCL have been the focus of tissue engineering researchers. Recent studies [217,218] have confirmed that changes in surface morphology directly affect the proliferation and differentiation behavior of cells growing on the scaffold, and that porous scaffolds with rough surfaces facilitate cellular osteogenic differentiation. In addition to focusing on improving PCL itself, its use in combination with one or more other biomaterials to enhance the induced osteogenic properties or biomechanical strength is also a promising avenue [219]. For example, combining PLC with ceramic calcium inorganic materials such as hydroxyapatite or tricalcium phosphate can improve its mechanical strength and osteogenic properties. Park et al. added β-TCP to 3D-printed PCL scaffolds for dental applications (Figure 5A). Incorporating a high β-TCP content into the composite scaffold increased the scaffold’s surface roughness, porosity, and wettability, promoting osteogenic differentiation and proliferation of mouse MSC lines [220]. Qu et al. [221] combined electrostatic spinning and additive manufacturing to fabricate PCL–nano–HA scaffolds. The scaffolds have precise micro-scale fiber orientation and alignment to better mimic the collagen fiber and HA crystal composites found in natural bone. This printing technique, called electrohydrodynamic printing, provides very organized high-performance scaffolds, with good biocompatibility, that promote cell proliferation and the alignment of MC3T3-E1 cells.

Kim et al. prepared a BGS-7/PCL bioglass composite scaffold using a 3D fusion process for printing (Figure 5B). The composite scaffold consisted of different mass fractions (20, 40, and 60 wt%) of bioglass with a well-aligned pore structure. The bioactivity and toughness of the composites were significantly enhanced with a BGS-7 concentration of 40%. In addition, the composite scaffolds showed significantly higher toughness compared to that of pure bioglass scaffolds with similar porosity [222].

Kolan et al. developed a PCL/bioactive borate glass composite scaffold using Pluronic F127 hydrogel as a cell suspension medium (Figure 5C). Pluronic is a poly(ethylene oxide)-poly(propylene oxide)-poly(ethylene oxide) (PEO-PPO-PEO) triblock copolymer in which the sol-gel transition occurs when the temperature is elevated to 20 °C (the lower critical gel temperature) or higher [223]. Therefore, Pluronic hydrogels are used as support materials in the fabrication of complex-shaped sections or porous structures. Optical microscopy evaluations showed high macro- and microporosities of this support, as well as its fast glass dissolution. In addition, the formation of a hydroxyapatite-like layer on the surface showed the bioactivity of the scaffold. Fathi et al. prepared PCL/BG-SrCo composite scaffolds using the FDM process [224]. The prepared composite scaffolds exhibited improved mechanical properties. In vitro MG-63 cell attachment assessments demonstrated that the solid scaffold had no adverse effects on osteoblast growth and proliferation. Bouyer et al. prepared PCL/graphene 3D-printed composite scaffolds using the FDM process [225]. The high concentration of graphene improved the mechanical properties of the scaffolds. However, graphene had an inhibitory effect on Saos-2 cells.

#### 4.2.5. PLGA Composite Scaffolds

**Figure 6:**
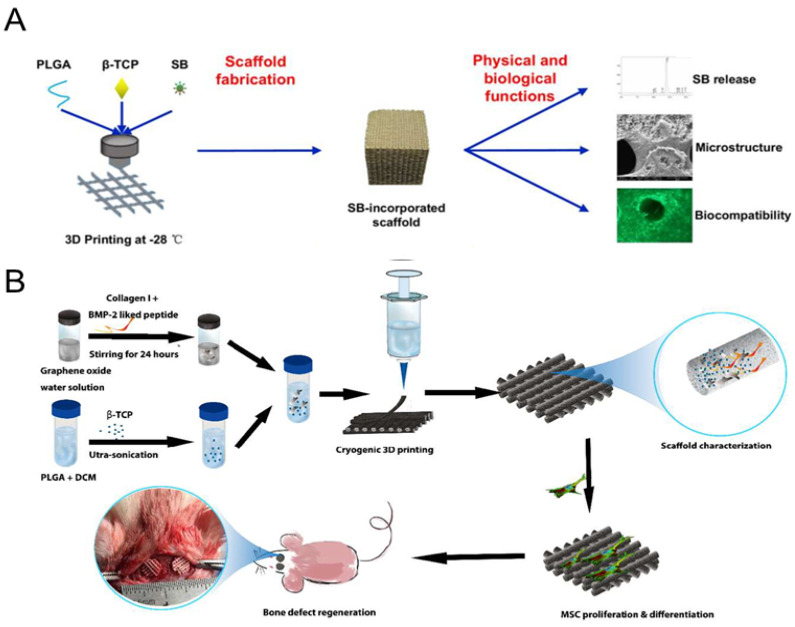
3The 3D-printed PGLA composite scaffolds. (**A**). The schematic diagram of low-temperature 3D printing technology combining PLGA/β-TCP scaffold with SB [229]. (**B**). The preparation of the peptide/GO/β-TCP/PLGA scaffold [231].

Poly(lactic-co-glycolic acid) copolymer (poly(lactic-co-glycolicacid), PLGA) is a biodegradable polymeric organic compound created by the random polymerization of two monomers, lactic acid and hydroxyacetic acid. PLGA has been approved by the US FDA since 1986 [226]. Poly(lactic-co-glycolic acid) copolymer (PLGA), also known as poly(lactic-co-glycolicacid), is a biodegradable organic compound composed of two monomers, lactic acid and glycolic acid, which undergo random polymerization. PLGA is widely used as an ideal sustained-release carrier due to its excellent biocompatibility and biodegradability. By adjusting the polymerization ratios and controlling the polymer length and speed, the release rate of PLGA can be tailored. Currently, PLGA is extensively utilized as a drug carrier and biological scaffold [227]. However, one limitation of synthesized PLGAs is their insufficient hydrophilicity, resulting in poor cell adhesion and the generation of acidic byproducts during degradation [228]. To overcome these challenges, PLGA is often combined with tricalcium phosphate (TCP) and collagen to fabricate composite scaffold materials that possess optimal acidity and biocompatibility for tissue growth and cell adhesion. Lin et al. employed low-temperature 3D printing to merge salvianolic acid B (SB) with PLGA/β-TCP (Figure 6A). Previous research has demonstrated that SB mitigates glucocorticoid-induced osteoporosis in rats by promoting osteogenesis and angiogenesis [229]. Moreover, it stimulates the metabolic activity of osteoblasts and alkaline phosphatase activity through the ERK signaling pathway [230]. However, SB’s chemical structure is susceptible to instability and oxidation. Incorporating SB into PLGA/β-TCP has been shown to enhance the osteogenesis and angiogenesis of the composite scaffold, thereby facilitating osseointegration [229].

Recent studies have demonstrated that PLGA/TCP and alginate/PLGA composite scaffolds, when loaded with BMP-2 and TGF-β1, exhibit promising capabilities for repairing osteochondral defects [232,233]. In the pursuit of further advancements, researchers have developed a novel bioactive PLGA/β-TCP composite scaffold using an innovative cryogenic 3D printing technique (Figure 6B), where graphene oxide (GO) and bone morphogenetic protein (BMP)-2-like peptide are incorporated in situ. This integration of graphene oxide not only enhances the compressive strength but also improves the surface wettability of the scaffolds. Moreover, the in situ loading strategy and the high adsorption capacity of graphene oxide nanosheets facilitate the sustained release of BMP-2, which proves advantageous for promoting bone growth, as well as facilitating the proliferation and adhesion of rMSCs [231].

**Table 2:** The 3D printed composite scaffolds for repairing bone defects.

Category		Disadvantages of a Single Material	Composite Materials	3D Printing Technology	3D Printing Equipment	Advantages	Application	Ref.
Bioceramic composite bone scaffolds	PMMA bone cement scaffold	Non-biological activity and hard to degrade, toxicity of MMA monomer, high modulus of elasticity	Chitosan/β-TCP/PMMA	3D laser drilling	100-W carbon dioxide (CO_2_) laser	Enhanced printability, heightened biological activity, non-toxic degradation byproducts, and favorable for osteoblast-like cell proliferation	Saos-2 cell culture	[234]
			TiO_2_/polyetheretherketone(PEEK)/PMMA	DLP	DLP Photocuring 3DP system	TiO2 enhanced antibacterial performance, while PEEK augmented mechanical strength and mitigated cytotoxicity	L929 cell culture	[235]
	CPC bone cement scaffold	Poor mechanical properties, slow degradation, not conducive to bone ingrowth	PLGA fiber/CPC	Extrusion-based 3DP	Custom-built extrusion-based 3D printer	PLGA fiber shortened the setting time of the CaP slurry, thereby enhancing the formability and shape fidelity of the CaP scaffold.	MG-63 cell culture	[173]
			CPC/VEGF hydrogel	3D plotting	InnoTERE GmbH	Improved control over the porosity and shape of CPC scaffolds	HDMEC cell culture	[177]
			Cap/alginic acid	Multi-channel 3D plotting	Fraunhofer IWS	Enhanced printability, improved mechanical properties and toughness, controlled release of proteins.	hMSCs cell culture	[175]
			PCL/CaP	Drop-on-demand printing (DDP)	A commercial DDP machine	Consistent printing with interconnected porous structure, high printing efficiency	HEPM cell culture	[176]
Polymer composite scaffolds	Chitosan hydrogel composite scaffolds	Poor mechanical properties, high swelling rate, and low solubility	Chitosan/cellulose nanofibers hydrogels	Extrusion-based printing (EBB)	An extrusion bioprinter with a 20 G nozzle	Superior printing ability with high-resolution printing structure, unique mechanical properties	NIH/3T3 cell culture	[195]
			HA/Chitosan/PVA hydrogels	EBB	Extrusion-based 3D printer	Elastic modulus similar to natural bone, excellent biocompatibility.	MSCs cell culture	[193]
			HA/Chitosan/Genipin	DIW	the Regemat V1 Hybrid printer	Improved rheology of bio-ink, enhanced mechanical properties	MG-63 cell culture	[188]
			Nanocellulose/chitosan	EBB	a BioAssemblyBot fitted with a 20 G nozzle	Promoted osteogenic effects of MC3T3-E1 cells by CNCs	MC3T3-E1 cell culture	[196]
	PLA composite scaffolds	High brittleness, poor toughness, slow degradation rate, and difficult surface modification	PLLA/nHA	FDM	FDM printer for 3DP	Low brittleness, reliable printing suitability, and accurate printing, high compressive strength	rMSCs cell culture	[212]
			FRTPs/PLA	FDM	a modified FlashForge printer	Excellent Young’s modulus and strength.	-	[216]
			PLA/BG	FDM	FDM 3D printer 2	PLA/BG composite scaffolds exhibited better biocompatibility and osteogenic properties compared to pure PLA and PLA/HA scaffolds	DPSCs cell culture	[213]
	PCL composite scaffolds	Non-biological activity, smooth surface, strong hydrophobicity, not suitable for osteoblast adhesion and bone tissue regeneration, poor mechanical strength, slow degradation	PCL/β-TCP	FDM	Lab-made 3D bioprinting system	The increase in β-TCP content led to increased surface roughness, porosity and wettability, and promoted cell growth and osteogenic differentiation of the non-cytotoxic D1 mouse mesenchymal stem cell line in vitro	D1-MSCs cell culture	[220]
			BGS-7/PCL	FDM	DASA-Robot system	PBGS-40 (40 wt% bioglass) composite scaffold had good toughness and reasonable cell viability	MC3T3-E1 cell culture	[222]
			PCL/bioactive borate glass	FDM	Assembled DIY 3D printer	High porosity, fast degradation rate	-	[223]
	PLGA composite scaffolds	Insufficient hydrophilicity, poor cell adhesion, acid degradation products	Salvianolic acid B/PLGA/β-TCP	Low-temperature rapid phrototyping (RP)	Low-temperature rapid-prototyping instrument	SB activates the ERK signaling pathway, thereby promoting osteogenesis and angiogenesis	Female SD rats in vivo	[229]
			Peptide/GO/β-TCP/PLGA	Cryogenic 3DP	Cryogenic 3DP machine	Enhanced compressive strength and surface wettability; continuous peptide release facilitates the proliferation and adhesion of rMSCs	Male Wistar rats in vivo	[231]

## 5. Conclusions and Perspective

This review presents an introductory exploration of the utilization of 3D printing technology in the production of bone repair scaffolds. In contrast to comparable articles of its kind, our review commences with a meticulous examination of scaffold material composition, centering on the intricacies of 3D printing techniques, 3D printing equipment for 3D-printed scaffolds, and efficacy pertaining to both single-material scaffolds and composite materials scaffolds.

The 3D fabricated scaffolds can be categorized into two distinct types, according to the materials employed: single-component scaffolds and composite scaffolds. The single-component scaffolds comprise five classifications: metallic biomaterial scaffolds, ceramic material scaffolds, BAG scaffolds, polymer scaffolds, and nanofiber scaffolds. Conversely, the composite scaffolds can be further segregated into seven divisions: PMMA bone cement scaffolds, CPC bone cement scaffolds, polymer scaffolds, gelatin scaffolds, PLA scaffolds, PCL scaffolds, and PLGA scaffolds. We have undertaken an extensive recapitulation and analysis of the material composition, 3D printing attributes, performance characteristics, advantages, disadvantages, and applications for each scaffold type. Additionally, predicated upon the present research status and progression, we have set forth recommendations for future investigations within this domain. In summary, this review serves as an invaluable point of reference for the advancement of research endeavors concerning bone repair scaffolds.

Based on the previous summary of the current status of 3D printed scaffolds for bone repair, it is not difficult to conclude that the future development directions for 3D printed bio-scaffolds for bone repair should be investigated, as follows:

(1) Material optimization and innovation

One of the important future research directions would be to further innovate and improve bone repair materials by exploring new biocompatible materials, such as biodegradable materials, natural polymers, and biomimetic materials, to enhance the biocompatibility and mechanical properties of scaffolds.

(2) Addressing tissue stability issues

In the process of bone repair, the stability of scaffolds is crucial. Future research should explore different forms of scaffold structures, such as porous structures, mesh structures, or layered structures, to promote cell growth and vascular regeneration and to improve the bonding strength and stability between the scaffold and surrounding tissues.

(3) Controlled drug release strategies and integration of bioactive substances

Firstly, controlled drug release is a key factor in bone repair. Developing precise and controllable drug delivery systems to achieve specific timing and dosage of drug delivery is vital. Nanomaterials, multilevel structures, or micropores can be used to control drug release and meet individualized treatment needs. Secondly, integrating bioactive substances (such as growth factors, drugs, and bioceramics) into scaffold materials, developing synergistic mechanisms between scaffold materials and bioactive substances, and optimizing their delivery and release methods should also be of prime importance.

(4) Bioprinting technology

Future research should explore higher precision and more complex bioprinting technologies to achieve fine bone tissue structures and morphologies. Researchers can use bioprinting technology to selectively print cells, growth factors, and other bioactive substances within the scaffold to promote faster bone regeneration and repair processes.

(5) Cell fusion and customization

Future research should explore methods to facilitate the fusion of scaffolds with cells and develop personalized design and fabrication methods based on individualized medical imaging and computational simulations. These methods can be used to develop customized scaffold designs and manufacturing techniques for each patient.
